# Association between Dietary Isoflavone Intake and Ulcerative Colitis Symptoms in Polish Caucasian Individuals

**DOI:** 10.3390/nu11081936

**Published:** 2019-08-17

**Authors:** Dominika Skolmowska, Dominika Głąbska, Dominika Guzek, Gustaw Lech

**Affiliations:** 1 Chair of Dietetics, Department of Dietetics, Faculty of Human Nutrition and Consumer Sciences, Warsaw University of Life Sciences (WULS-SGGW), 159c Nowoursynowska Str., 02-776 Warsaw, Poland; 2 Chair of Consumption Research, Department of Organization and Consumption Economics, Faculty of Human Nutrition and Consumer Sciences, Warsaw University of Life Sciences (WULS-SGGW), 159c Nowoursynowska Str., 02-776 Warsaw, Poland; 3 Department of General, Gastroenterological and Oncological Surgery, Medical University of Warsaw, 1a Banacha Str., 02-097 Warsaw, Poland

**Keywords:** ulcerative colitis, remission, isoflavones, daidzein, fecal mucus

## Abstract

Currently there are contradictory observations regarding the associations between the isoflavone intake and inflammatory bowel disease in terms of its prevention and treatment, and this may be attributed to the diversity of applied doses and influence of various isoflavones. The aim of the presented cross-sectional study is to analyze the association between intake of various isoflavones (daidzein, genistein, glicytein and total isoflavones) and ulcerative colitis symptoms (fecal blood, mucus and pus) in Polish Caucasian individuals in confirmed remission. Assessment of diet was based on self-reported data obtained from patients’ three-day dietary records and their individual assessments of symptoms. A total of 56 Caucasian patients with ulcerative colitis in confirmed remission were recruited for the study (37 females and 19 males, aged 18–80). For individuals with no fecal mucus observed, higher daidzein (*p* = 0.035, 122 vs. 19 µg) and total isoflavone intakes (*p* = 0.034, 302.2 vs. 123.7 µg) were observed in comparison with individuals not declaring this symptom, while for daidzein it was confirmed for the component density of their diets. The opposite association was stated for fecal pus, as for individuals with a lack of this symptom, lower daidzein intake was stated in comparison with individuals declaring this symptom (*p* = 0.049, 103.3 vs. 206.7 µg), but it was not confirmed for the component density of the diets. It was stated that the high intake of isoflavones by Caucasian individuals, as in a western diet, may influence the symptoms of ulcerative colitis, with the strongest influence by daidzein. Taking this into account, isoflavones may be included into the diets of ulcerative colitis patients in remission if well-tolerated, but there is a need for further study.

## 1. Introduction

Ulcerative colitis is a chronic idiopathic inflammatory disease affecting the colonic mucosa that typically extends proximally from the rectum [1]. The clinical course of ulcerative colitis is unpredictable and involves alternating periods of remission and exacerbation [2]. However, the exact etiology of the disease remains unclear, and the primary attributable causes are genetic predisposition, dysregulated immune response and environmental factors [3]. Although the symptoms depend on the severity and extent of the disease, one of the most frequent symptoms is diarrhea, which may be accompanied by observable blood, mucus or pus contents [4], and may be observed even in remission [5].

As the occurring symptoms can significantly influence the health-related quality of life, various treatment methods are applied to relieve them and to prevent exacerbations and further complications [6,7]. Although medical therapy poses the first-line therapy for managing ulcerative colitis, diet is gaining significant interest as a non-pharmacological approach to manage the disease [8]. Moreover, diet has been speculated as an important factor for both the pathogenesis and therapy of ulcerative colitis [9,10]. Although there are no evidence-based dietary guidelines for patients with inflammatory bowel diseases, some components are considered to play a significant role in influencing the course of the disease [11].

Among various components, isoflavones have been indicated as a potential factor for the prevention and treatment of inflammatory bowel diseases due to their anti-inflammatory effect [12]. This includes the inhibition of reactive nitrogen species generation, leukocyte migration and nuclear factor kappa B (NF-κB) activity and reduction of pro-inflammatory mediators production [13]. However, despite increasing interest, there is a lack of plausible scientific evidence between intake of isoflavones and ulcerative colitis symptoms [14]. So far no clinical trials have been performed in association with the effect of isoflavones on ulcerative colitis; rather, there have been mainly animal model studies and some sparse descriptive human studies.

On the one hand, in the experimental models of inflammatory bowel disease, soy isoflavones have inhibited induced colitis and reduced crypt damage [15,16]. On the other hand, in European populations, there have not been many studies on ulcerative colitis patients, and the results have been contradictory. This suggests that the intake of various isoflavones may result in either beneficial or adverse effects on certain symptoms of the disease [17] caused by their various mechanisms of action [18]. Moreover, among Asian populations, high isoflavone intake (much higher than for European populations) is indicated to be associated with the development of the disease [19], suggesting that both studied isoflavone and applied dosage may significantly influence the observed effect. It is emphasized that some preclinical evidence suggests that isoflavones have the potential to improve the prognosis of inflammatory bowel diseases [20]. Taking this into account, there is need to assess the various subclasses in order to translate the current knowledge from preclinical models into further studies and dietary recommendations [12].

The aim of the present study is to analyze the association between the intake of isoflavones (daidzein, genistein, glicytein and total isoflavone) and ulcerative colitis symptoms (fecal blood, fecal mucus and fecal pus) in Polish Caucasian individuals in confirmed remission.

## 2. Materials and Methods

### 2.1. Study Design

The cross-sectional study of the Polish Caucasian ulcerative colitis individuals was carried out at the Dietetic Outpatient of the Department of Dietetics, Warsaw University of Life Sciences (WULS-SGGW). The study was conducted in compliance with the guidelines presented in the Declaration of Helsinki, and all procedures related to human subjects received approval of the Bioethical Commission of the Central Clinical Hospital of the Ministry of Interior in Warsaw (No. 35/2009) and the Bioethical Commission of the National Food and Nutrition Institute in Warsaw (No. 1604/2009).

### 2.2. Study Participants

The study was conducted among Polish female and male individuals with diagnosed ulcerative colitis in confirmed remission; the group was recruited from the Warsaw Gastroenterology Outpatient Clinics (i.e., the Gastroenterology Outpatient Clinic at the Central Clinical Hospital of the Ministry of Interior in Warsaw; the Gastroenterology Outpatient Clinic at the Public Central Teaching Hospital in Warsaw; and the Gastroenterology Outpatient Clinic at the Maria Skłodowska-Curie Memorial Cancer Centre, Institute of Oncology). The participants were recruited in a procedure of network convenience sampling with a snowball effect, in which they were invited by their gastroenterologists to participate. A total of 56 individuals with ulcerative colitis in confirmed remission (37 females and 19 males), aged 18–80, were included for the study.

Inclusion criteria were as follows:

1) free-living patients;
2) Caucasian;
3) age 18–80 years;
4) ulcerative colitis diagnosed after endoscopic examination;
5) confirmed clinical remission lasting for at least 6 weeks, assessed based on the 6-point Mayo Score (as a method indicated as a validated marker of ulcerative colitis activity that has been recommended for use in clinical trials and clinical practice [21] and is highly correlated with the full Mayo score [22]) and the Rachmilewitz index for assessment of ulcerative colitis activity;
6) confirmed endoscopic remission (image with no changes or disappearance of the vascular network, erythema, inflammatory polyps allowed) for routine endoscopy performed over a period of last 6 weeks;
7) constant dose of drugs for at least 6 weeks;
8) written informed consent to participate in the study.

The detailed characteristics of the studied group, including age, Body Mass Index (BMI), treatment duration and concurrent diseases, were presented in our previous publication, where it was stated that they did not differ between male and female participants [23]. The basic characteristics of the disease course for the studied group are presented in Table 1.

To assess their ulcerative colitis symptoms, an interview about their subjectively perceived situation, in comparison with their situation before the diagnosis, was undertaken [24]. The participants of the study were asked about either the lack or presence of fecal blood, fecal mucus and fecal pus, which in ulcerative colitis may be observed even in remission [5]. The structured questionnaire was applied with the questions about the previous (before the ulcerative colitis diagnosis) and current (after diagnosis, but exactly in the period of remission) intensity and frequency of fecal blood, fecal mucus and fecal pus. Before completing the questionnaire, participants were directed to base their declarations only on their own and real observations, not on any suppositions and not on any results of medical examinations. They were also asked if they required a description of fecal blood, fecal mucus and fecal pus presented and, if they stated that they did required it, an image of typical feces with blood/mucus/pus was verbally described. Afterwards, participants were directed to compare the current intensity and frequency of these symptoms with those before the ulcerative colitis was diagnosed. A lack of fecal blood, mucus or pus in the period of remission, or no increase in the intensity or frequency of the abovementioned symptoms compared to those that were observed before the ulcerative colitis diagnosis, was interpreted as a lack of these symptoms. Afterwards, patients were stratified into sub-groups based on their lack or presence of symptoms.

### 2.3. Diet Analysis

An assessment of diet was based on self-reported data from the participants’ dietary records obtained over a period of three random and typical days (two weekdays and one weekend day), as is commonly applied. The dietary record method was chosen, as it is stated to be a “gold standard” [25], and is recommended by the Food and Agriculture Organization of the United Nations (FAO) [26]. To receive the reliable and precise assessment of food intake, all patients were informed about the rules of dietary record and about the necessity of the scrupulous recording of all consumed food products and beverages. The serving sizes were checked using the Polish Atlas of Food Products and Dishes Portion Size [27]. The energy value of diet (kcal) was analyzed using Polish dietician software (Dietetyk 2.0) with the Polish database of the nutritional value of the products [28].

Due to the lack of a Polish database of isoflavones in food products, their intake was evaluated using the National Nutrient Database for Standard Reference of the United States Department of Agriculture (USDA) [29]. In the USDA database there is information about 281 food products containing isoflavones of the specified amount, and 271 food products are recognized as having zero values (which is defined as a situation in which the analytical method did not find isoflavones in products and reported them as not detected). The intake of the following components was calculated: daidzein (µg), genistein (µg), glicitein (µg) and total isoflavones (µg). As isoflavone content depends on thermal processing, different forms of the same food products were taken into account (e.g., raw, dried, boiled, cooked). Then, further analysis was conducted based on the average intake of the abovementioned components (mean value from three recorded days).

The intake levels of isoflavones were compared between the sub-groups of patients that were stratified based on them declaring or not declaring the symptoms of ulcerative colitis (fecal blood, mucus and pus). The intake of isoflavones was presented in µg and calculated per 1000 kcal of diet (µg/1000 kcal) to determine the component density of diets.

### 2.4. Statistical Analysis

The obtained data are presented as mean ± SD values with minimum, maximum and median values indicated. The distribution of analyzed factors were verified using a Shapiro–Wilk test and, because of the non-parametric distributions observed, the differences between two groups were identified using a Mann–Whitney U test. The accepted level of significance was set at *p* ≤ 0.05. Statistical analysis was carried out using Statistica software version 8.0 (StatSoft Inc., Tulsa, OK, USA).

## 3. Results

The isoflavone intakes in groups of individuals with ulcerative colitis declaring either lack or presence of fecal blood are presented in Table 2. Fecal blood was claimed by 69% of the analyzed patients with ulcerative colitis. No differences were observed in comparison of isoflavone intake between groups.

The isoflavone intakes per 1000 kcal in groups of individuals with ulcerative colitis declaring either lack or presence of fecal blood are presented in Table 3. No differences were observed in comparison of isoflavone intake per 1000 kcal between groups.

The isoflavone intakes in groups of individuals with ulcerative colitis declaring either lack or presence of fecal mucus are presented in Table 4. Fecal mucus was claimed by 16% of the analyzed patients with ulcerative colitis. Patients who did not declare fecal mucus had at the same time a higher intake of daidzein (122 µg per day, which varied from 4.3 to 977.7 µg) compared to patients who declared fecal mucus (19 µg per day, which varied from 3.3 to 353 µg; *p* = 0.035). Furthermore, patients who did not declare fecal mucus had a higher total isoflavone intake (302.2 µg per day, which varied from 27.7 to 1574 µg) compared to patients who declared fecal mucus (123.7 µg, which varied from 18 to 4417 µg; *p* = 0.034). No differences were observed in comparison of other isoflavone intakes between groups.

The isoflavone intakes per 1000 kcal in groups of individuals with ulcerative colitis declaring either lack or presence of fecal mucus are presented in Table 5. Patients who did not declare fecal mucus had at the same time a higher intake of daidzein (62.3 µg per 1000 kcal, which varied from 1.3 to 392.7 µg) compared with patients who declared fecal mucus (12.1 µg per 1000 kcal, which varied from 2.1 to 347.7 µg; *p* = 0.032). No differences were observed in comparison of other isoflavone intakes between groups.

The isoflavone intakes in groups of individuals with ulcerative colitis declaring either lack or presence of fecal pus are presented in Table 6. Fecal pus was claimed by 5% of the analyzed patients with ulcerative colitis. Patients who declared fecal pus had a higher intake of daidzein (206.7 per day, which varied from 205.7 to 353 µg) compared with patients who did not declare fecal pus (103.3 µg per day, which varied from 3.3 to 977.7 µg; *p* = 0.049). No differences were observed in comparison of other isoflavone intakes between groups.

The isoflavone intakes per 1000 kcal in groups of individuals with ulcerative colitis declaring either lack or presence of fecal pus are presented in Table 7. No differences were observed in comparison of isoflavone intakes between groups.

## 4. Discussion

In this study, which was conducted among a group of individuals with ulcerative colitis, the most important observation was that patients declaring a lack of fecal mucus were characterized by a higher intake of daidzein and higher total isoflavone intake compared with patients declaring the presence of fecal mucus. It is well known that a layer of mucus protects the intestinal surface [13]. However, in cases where the mucosal layer is damaged by inflammation, mucus is excreted in the stool, which is one of the symptoms of the inflammatory process [30]. This has been commonly observed in ulcerative colitis, and is the most important symptom, indicating the impending worsening of symptoms [31] as its composition and characteristics change with the progression of the disease [32]. Moreover, the mucus layer degradation, which may be observed in exacerbation, is associated with the opening of the intestinal barrier for microbial intestine aggression, thus resulting in the progression of the disease [33]. The presence of visible mucus in the stool is stated primarily in exacerbation, so in sub-groups in the presented study declaring this symptom, it may be interpreted as a generally worsened condition in patients, with a higher possibility of exacerbation in the near future.

It may be suggested that the beneficial effect of isoflavones, which contribute to reducing the severity of inflammation caused by a reduction in the production of pro-inflammatory cytokines, restores the balance of colonic mucosa [34]. In animal model studies, this has been observed for the influence on secretion and/or expression of tumor necrosis factor-α [16,35,36,37,38], NF-κB [38], interleukin (IL)-1β [35], IL-6 [34,36], IL-12p40 [34], IL-17a [34], IL-18 [36], interferon-γ [34,35,36], cyclooxygenase-2 [39,40] and inducible nitric oxide synthase [16,41]. Such a mechanism may explain the beneficial effect of isoflavones, especially daidzein, on the presence of fecal mucus.

Patients who were affected by fecal pus were characterized by a higher intake of daidzein; thus, it may be indicated that, for this symptom, isoflavones may have a negative impact. Therefore, based on the obtained results, it cannot be undoubtedly determined whether the influence of isoflavone intake on recurring ulcerative colitis symptoms is positive or negative. The specific influence of isoflavones that was stated for pus, with the opposite effect for mucus, may result from the role, visibility and frequency of pus and mucus in individuals with ulcerative colitis. Generally, pus is indicated as one of the descriptors of exacerbation [42], and its presence in the intestine is indicated as one of the determinants of the status of mucosa [43], and may allow the diagnosis of endoscopic exacerbation. Moreover, the presence of pus may be associated with active inflammation [44]. However, in general, observable fecal pus is a rare symptom that in the presented study was stated by only three patients. Moreover, it is commonly known that fecal pus is hardly recognizable to the naked eye [45]; therefore, it may be possible that the participants who reported pus content in their stool may have misdiagnosed their mucus or loose stool without properly digested elements as pus content. In spite of the indicated issues, with no specific evidence in the studied group, it cannot be concluded that the association between isoflavone intake and fecal pus did not exist. Taking it into account, based on the obtained results, it should be supposed that higher daidzein and total isoflavone intakes combined may promote a lack of fecal mucus, but that higher daidzein intake alone may promote fecal pus.

Soy isoflavones, such as genistein and daidzein, have an estrogen-like activity [46]. Due to this activity, it may be supposed that high isoflavone intake could play a role in the pathogenesis of ulcerative colitis. This effect may be caused by a similar mechanism of action as that for estrogens, which involves estrogen binding to the β-estrogen receptor in the intestine and contributes to the modification of the mucosal barrier; consequently, it may lead to an abnormal immune response [19,47]. When analyzing the literature, it may be concluded that there are premises that high isoflavone intake may be a factor that is significantly related to the risk of ulcerative colitis development [19].

In animal model studies, it seems that the observed effect of isoflavone intake may be dose-dependent and dependent on other factors, including microbiota and individual predisposition. In the study conducted on mice, isoflavone intake showed a beneficial effect, thus reducing the severity of inflammation in the large intestine when daidzein-rich isoflavone aglycones were administered at doses of 100,000 µg per kilogram of body weight, containing 80,000 µg isoflavones per kilogram of body weight [34]. However, when comparing daidzein, genistein and equol (being the bacterial metabolite of daidzein) at lower doses (up to 20,000 µg per kilogram of body weight), it has been noted that while daidzein and genistein do not have a significant influence, equol at a dosage of 20,000 µg per kilogram of body weight can result in the exacerbation of induced colitis in mice [48]. For the mentioned influence of equol, as a metabolite of daidzein, it must be indicated that it is generated by fecal microbiota metabolism in only 25–30% of adults in Western countries [49], but by 60% of adults in Asian countries [50].

The only study conducted in Japan identified the negative influence of isoflavones, presenting a higher intake of daidzein and genistein in the pre-disease period in ulcerative colitis individuals compared to healthy controls [19]. This specific influence may be partly explained by the fact that a high isoflavone intake in Asian population may cause excessive equol generation. However, it must be emphasized that the average isoflavone intake varies for different nationalities [51]. The Japanese diet is characterized by a high content of these compounds because of the high consumption of soy and fermented soy products in that country. Consequently, Japanese individuals consume an average of 25,000–50,000 µg of isoflavones a day [52], while European individuals consume <1000 µg of these compounds daily [53].

Therefore, the amount of isoflavones, which is generally consumed by patients with ulcerative colitis in European countries, might be below the range in which a positive effect on the disease symptoms is observed, and only a high European intake, in the current study, was associated with reduced fecal mucus. On the contrary, it may be supposed that the high intake observed in the Japanese study by Ohfuji et al. [19] was too high to exert a positive influence, and contributed to an increased risk of the disease.

While the form-dependent and dose-dependent effect of isoflavone is putative, the dose should be specified for each form of isoflavones. Currently there are no specific guidelines or recommendations concerning isoflavone intake in patients with diagnosed ulcerative colitis, while for healthy individuals they have also not been formulated.

The major sources of isoflavones are legume seeds, such as soy, beans, peas and lentils; however, small amounts of these compounds are found in cereals, vegetables and fruits [29]. Nevertheless, legume seeds contain high amounts of fiber and oligosaccharides, which are not digested by human enzymes [54]; consequently, some people experience increased flatulence while consuming legume seeds [55]. This is crucial, especially for individuals with ulcerative colitis, because bloating is an often-reported symptom of this group [56].

Although diet is a behavioral factor that is primarily manipulated by patients with ulcerative colitis to improve their physical well-being and to maintain remission [57], patients try to apply different dietary strategies to relieve gastrointestinal symptoms [58], and so such recommendations should be formulated. However, it may be assumed that isoflavones may be included in the western diet in moderate amounts if they are well tolerated.

In spite of this fact that some interesting differences associated with isoflavone intake have been observed, the limitations of this study must be also presented. The main factor limiting the possibility of a conclusion is the fact that the presented study was a cross-sectional one, and no planned dietary intervention, in the case-control study, has so far been conducted to assess the influence of isoflavone intake in ulcerative colitis individuals. Moreover, while fecal blood was quite common in the studied group of patients in remission, both mucus and pus were very rare, so a small number of participants declaring those symptoms disenabled multi-factor analysis, causing the sample size to be indicated as the other limitation. Furthermore, the fact that the assessed group was quite heterogenic for its gender, age, BMI and concurrent diseases may be indicated as another interfering factor. The nutritional analysis, based on the food composition tables (with no direct analysis of the composition of consumed food products) has been also an important bias in all nutritional studies, as nutritional databases present only typical content of components, and not the content consumed by a specific patient. Especially if there are no national databases, this may be indicated as a limitation. Last, but not least, the conducted analysis was based on the subjective assessment of patients, which for them is very important and influences their well-being, but is another important factor that may have limited our conclusions.

## 5. Conclusions

It may be concluded that, in Caucasian individuals suffering from ulcerative colitis, the high intake of isoflavones in a Western diet may influence symptoms, and that for daidzein the influence is the strongest. Higher daidzein and total isoflavone intakes may promote a lack of fecal mucus. At the same time, it may be supposed that higher daidzein alone may promote fecal pus; however, there is a need for further studies concerning the relationship between isoflavone intake and ulcerative colitis symptoms in remission.

## Figures and Tables

**Table 1:** The basic characteristics of the disease course for the studied group.

Variable		Individuals (*n* = 56)
Duration of the ulcerative colitis	<4 years	8 (14.2%)
	4–6 years	24 (42.8%)
	7–9 years	13 (23.2%)
	>9 years	11 (19.8%)
Number of groups of applied medicines *	1 group	40 (71.4%)
	2 groups	11 (19.6%)
	3 groups	5 (10.0%)
Number of bowel movements per day	1	1 (1.8%)
	2	20 (35.7%)
	3	13 (23.2%)
	4	15 (26.8%)
	>4	7 (12.5%)
Mean number of hospitalizations per year	0	12 (21.4%)
	0.1–0.3	15 (26.9%)
	0.31–0.49	17 (30.3%)
	>0.5	12 (21.4%)

* in the groups of 5-aminosalicylic acid medications, corticosteroid medications and immunosuppressive medications.

**Table 2:** The isoflavone intakes in groups of individuals with ulcerative colitis declaring either lack or presence of fecal blood.

Intake of Isoflavones	Individuals Declaring Lack of Fecal Blood (*n* = 17)		Individuals Declaring Fecal Blood (*n* = 39)		*p*-Value **
	Mean ± SD	Median (min–max)	Mean ± SD	Median (min–max)	
Daidzein (µg)	175.3 ± 227.6	124.3 (12.0–977.7) *	131.1 ± 108.5	103.3 (3.3–476.7) *	0.682
Genistein (µg)	135.2 ± 108.6	156.7 (5.3–359.3)	195.9 ± 249.0	126.7 (2.3–1428.3) *	0.688
Glicytein (µg)	4.0 ± 13.8	0.0 (0.0–56.0) *	0.9 ± 5.3	0.0 (0.0–33.3) *	0.369
Total isoflavones (µg)	314.6 ± 269.0	302.7 (27.7–1137.7) *	328.0 ± 292.1	256.7 (18.0–1574.0) *	0.880

* nonparametric distribution (verified using Shapiro–Wilk test; *p* ≤ 0.05); ** compared using Mann–Whitney U test (due to nonparametric distribution).

**Table 3:** The isoflavone intakes per 1000 kcal in groups of individuals with ulcerative colitis declaring either lack or presence of fecal blood.

Intake of Isoflavones	Individuals Declaring Lack of Fecal Blood (*n* = 17)		Individuals Declaring Fecal Blood (*n* = 39)		*p*-Value **
	Mean ± SD	Median (min–max)	Mean ± SD	Median (min–max)	
Daidzein (µg/1000 kcal)	92.9 ± 103.6	65.2 (6.5–392.7) *	71.6 ± 74.2	59.4 (1.3–347.7) *	0.650
Genistein (µg/1000 kcal)	75.5 ± 74.4	61.7 (1.6–279.5) *	102.2 ± 137.5	69.1 (1.4–759.2) *	0.670
Glicytein (µg/1000 kcal)	2.7 ± 9.1	0.0 (0.0–37.4) *	0.5 ± 2.8	0.0 (0.0–17.7) *	0.368
Total isoflavones (µg/1000 kcal)	171.1 ± 146.0	135.8 (8.3–503.9) *	174.4 ± 171.5	128.6 (8.6–836.7) *	0.901

* nonparametric distribution (verified using Shapiro–Wilk test; *p* ≤ 0.05); ** compared using Mann–Whitney U test (due to nonparametric distribution).

**Table 4:** The isoflavone intakes in groups of individuals with ulcerative colitis declaring either lack or presence of fecal mucus.

Intake of Isoflavones	Individuals Declaring Lack of Fecal Mucus (*n* = 47)		Individuals Declaring Fecal Mucus (*n* = 9)		*p*-Value **
	Mean ± SD	Median (min–max)	Mean ± SD	Median (min–max)	
Daidzein (µg)	155.8 ± 157.9	122.0 (4.3–977.7) *	85.5 ± 120.0	19.0 (3.3–353.0) *	0.035
Genistein (µg)	195.6 ± 231.4	145.0 (2.3–1428.3) *	82.8 ± 68.8	81.7 (6.7–214.3)	0.074
Glicytein (µg)	2.3 ± 9.6	0.0 (0.0–56.0) *	0.0 ± 0.0	0.0 (0.0–0.0) *	0.382
Total isoflavones (µg)	353.7 ± 293.0	302.3 (27.7–1574.0) *	168.4 ± 156.6	123.7 (18.0–441.7)	0.034

* nonparametric distribution (verified using Shapiro–Wilk test; *p* ≤ 0.05); ** compared using Mann–Whitney U test (due to nonparametric distribution).

**Table 5:** The isoflavone intakes per 1000 kcal in groups of individuals with ulcerative colitis declaring either lack or presence of fecal mucus.

Intake of Isoflavones	Individuals Declaring Lack of Fecal Mucus (*n* = 47)		Individuals Declaring Fecal Mucus (*n* = 9)		*p*-Value **
	Mean ± SD	Median (min–max)	Mean ± SD	Median (min–max)	
Daidzein (µg/1000 kcal)	81.4 ± 78.5	62.3 (1.3–392.7) *	60.8 ± 111.6	12.1 (2.1–347.7) *	0.032
Genistein (µg/1000 kcal)	102.3 ± 130.0	69.1 (1.4–759.2) *	51.1 ± 40.9	52.2 (3.2–115.1)	0.220
Glicytein (µg/1000 kcal)	1.4 ± 6.1	0.0 (0.0–37.4) *	0.0 ± 0.0	0.0 (0.0–0.0) *	0.382
Total isoflavones (µg/1000 kcal)	185.1 ± 166.7	137.2 (8.3–836.7) *	111.9 ± 132.6	68.4 (8.6–435.0) *	0.078

* nonparametric distribution (verified using Shapiro–Wilk test; *p* ≤ 0.05); ** compared using Mann–Whitney U test (due to nonparametric distribution).

**Table 6:** The isoflavone intakes in groups of individuals with ulcerative colitis declaring either lack or presence of fecal pus.

Intake of Isoflavones	Individuals Declaring Lack of Fecal Pus (*n* = 53)		Individuals Declaring Fecal Pus (*n* = 3)		*p*-Value **
	Mean ± SD	Median (min–max)	Mean ± SD	Median (min–max)	
Daidzein (µg)	138.3 ± 155.0	103.3 (3.3–977.7) *	255.1 ± 84.8	206.7 (205.7–353.0) *	0.049
Genistein (µg)	178.7 ± 223.0	135.0 (2.3–1428.3) *	156.3 ± 86.4	126.7 (88.7–253.7)	0.771
Glicytein (µg)	2.0 ± 9.0	0.0 (0.0–56.0) *	0.0 ± 0.0	0.0 (0.0–0.0) *	0.654
Total isoflavones (µg)	319.0 ± 289.9	256.0 (18.0–1574.0) *	411.4 ± 68.2	441.7 (333.3–459.3)	0.167

* nonparametric distribution (verified using Shapiro–Wilk test; *p* ≤ 0.05); ** compared using Mann–Whitney U test (due to nonparametric distribution).

**Table 7:** The isoflavone intakes per 1000 kcal in groups of individuals with ulcerative colitis declaring either lack or presence of fecal pus.

Intake of Isoflavones	Individuals Declaring Lack of Fecal Pus (*n* = 53)		Individuals Declaring Fecal Pus (*n* = 3)		*p*-Value **
	Mean ± SD	Median (min–max)	Mean ± SD	Median (min–max)	
Daidzein (µg/1000 kcal)	72.4 ± 77.4	52.6 (1.3–392.7) *	178.8 ± 146.3	95.1 (93.6–347.7) *	0.081
Genistein (µg/1000 kcal)	94.5 ± 125.1	61.7 (1.4–759.2) *	87.3 ± 30.0	87.3 (57.4–117.4)	0.560
Glicytein (µg/1000 kcal)	1.2 ± 6.0	0.0 (0.0–37.4) *	0.0 ± 0.0	0.0 (0.0–0.0) *	0.654
Total isoflavones (µg/1000 kcal)	168.1 ± 163.3	129.7 (8.3–836.7) *	266.2 ± 149.4	212.5 (149.4–151.0)	0.136

* nonparametric distribution (verified using Shapiro–Wilk test; *p* ≤ 0.05); ** compared using Mann–Whitney U test (due to nonparametric distribution).
